# Iatrogenic withdrawal syndrome in adult intensive care unit: a scoping review

**DOI:** 10.3389/fmed.2025.1573363

**Published:** 2025-07-23

**Authors:** Emanuel Moisa, Georgeana Tuculeanu, Dan Corneci, Silvius Ioan Negoita, Federico Bilotta

**Affiliations:** ^1^3rd Department of Anesthesiology and Critical Care, Carol Davila University of Medicine and Pharmacy, Bucharest, Romania; ^2^Department of Anesthesiology and Critical Care, Elias Emergency University Hospital of Bucharest, Bucharest, Romania; ^3^Department of Anesthesiology and Critical Care, Dr. Carol Davila Central Military Emergency University Hospital, Bucharest, Romania; ^4^Department of Anesthesiology and Critical Care, University of Rome La Sapienza, Rome, Italy

**Keywords:** iatrogenic withdrawal syndrome, opioid, benzodiazepine, hypnotics, dexmedet- omidine, clonidine, sedation

## Abstract

**Introduction:**

Following prolonged opioid and/or hypnotic exposure, iatrogenic withdrawal syndrome (IWS) can develop in critically ill patients due to improper cessation of these drugs. While IWS is well-documented in pediatric and neonatal intensive care unit (ICU), research on adult ICU patients remains scarce. This scoping review aimed to map existing evidence on IWS in critically ill adults, focusing on diagnosis, epidemiology, risk factors, complications, clinical effects, treatment, and prevention.

**Methods:**

A literature search across PubMed, Scopus, and Web of Science included studies from 1990 to 2024 with prospective, retrospective, or randomized controlled trial designs. Out of 3105 retrieved titles, 29 studies met inclusion criteria.

**Results:**

Most studies addressed diagnosis (83%) and epidemiology (79%), with IWS definitions largely adapted from chronic drug users. Incidence varied from 13.6 to 49.5%. Several studies identified risk factors, primarily therapy-related, but only some performed robust statistical analyses. Complications and clinical effects were discussed in 12 studies but results on ICU and hospital outcomes were inconsistent. Physiological studies linked IWS to sympathetic overactivity and central nervous system excitability. Only 20% of studies examined treatment or prevention, with randomized trials assessing substitution therapy. Most strategies did not significantly alter IWS incidence, though clonidine showed potential benefits.

**Discussion:**

This review highlights critical knowledge gaps and the lack of consensus or guidelines for IWS in adult ICU patients, emphasizing the need for further research.

## Graphical Abstract

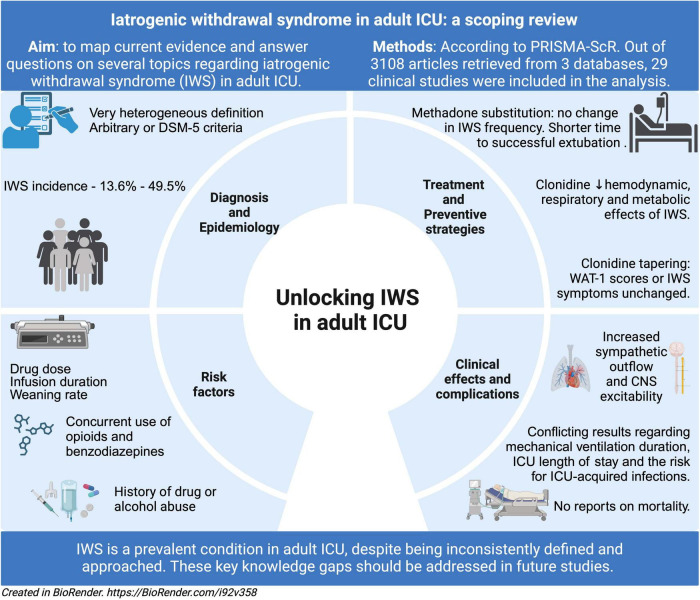

## Introduction

Iatrogenic withdrawal syndrome (IWS) refers to a condition typical of chronic opioid or hypnotic use, which can develop in critically ill patients following abrupt cessation or inadequate tapering of at least one drug, in either chronic abusers or drug-naïve patients (1). The neurobiology of IWS is not well-documented. Nonetheless, it involves a common pathway leading to increased central nervous system excitability and sympathetic outflow. As a result, patients exhibit a plethora of multisystemic signs and symptoms which complicates the management of critical illness (1, 2).

A significant amount of literature has been dedicated to withdrawal associated with chronic opioid and benzodiazepine use (3), as well as to IWS in neonatal and pediatric intensive care unit (ICU) (4). Nevertheless, evidence on IWS in adult critically ill patients remains scarce, precluding physicians from undertaking appropriate screening, diagnostic, prophylactic and therapeutic strategies in front of this condition (5, 6).

Available reports suggests that various critically ill patient subgroups could exhibit inherent (7–9) or ICU-related (8, 10, 11) risk factors for IWS. Some of these are modifiable (i.e., therapy-related aspects (8, 10, 11)) and are worth of more in-depth research in order to develop potential preventive strategies. Hence, a better understanding of this condition could eventually impact patients’ outcomes, such as ICU length of stay (LOS) (12), mechanical ventilation duration (12) and ICU-acquired infections rates (13).

As IWS is an emerging and overlooked topic, represented by a heterogeneous body of literature, a scoping review can cover a broad range of data and answer multiple research questions. Featuring a more flexible framework, it allows the selection of various study designs, emphasizing existing research gaps on IWS.

The aim of this scoping review is to map current evidence on diagnosis, epidemiology, risk factors, clinical effects, complications, treatment and preventive strategies for IWS in patients exposed to opioids, hypnotics and α-2 agonists.

## 2 Methods

This scoping review was conducted in accordance with the Preferred Reporting Items for Systematic Reviews and Meta-analyses for scoping reviews (PRISMA-ScR) guidelines (14) (PRISMA-ScR Checklist available in Supplementary material 1).

### 2.1 Search strategy

The search strategy was based on a combination of keywords from 3 separate fields: (i) drug used for analgesia and/or sedation; (ii) withdrawal and (iii) ICU (population/setting). Dedicated search strings were applied for the following databases: PubMed, Scopus and Web of Science. Database search was conducted by 2 independent reviewers (G.T., S.N.) and the complete search strategy is available in Supplementary material 2.

### 2.2 Inclusion and exclusion criteria

Articles were included in the analysis if: (i) were published between 01.01.1990 and 30.09.2024, (ii) were designed as observational prospective or retrospective cohort studies or as randomized controlled trials (RCTs), (iii) the studied population was from ICU, (iv) the primary and/or secondary outcomes were related to at least one research question (defined later). Articles were excluded if: (i) non-human population; (ii) basic science studies, (iii) pediatric or neonatal population; (iv) non-ICU patients, (v) case series, case report, qualitative studies, editorial, letter to the editor, book chapter, abstracts, literature review, dissertation (vi) other language than English. No geographical or sex restriction was applied.

### 2.3 Study selection

Study selection was performed using the Rayyan online platform (an artificial intelligence online platform developed for systematic literature search strategies) (15). A 95% duplicate exclusion criterion was applied after references from all searched databases were introduced in the Rayyan platform. The remaining duplicates were manually removed. Two independent reviewers (G.T., S.N.) screened the reference list after deduplication based on article title and abstract as a first screening phase. Articles considered to meet the eligibility criteria were subsequently assessed in a second phase based on their full text, by two independent reviewers (E.M., D.C.). Lastly, each reviewer introduced the articles for the final analysis in an electronic file and potential disagreements were resolved by consensus.

### 2.4 Data extraction

After article selection procedure was performed, the following data were extracted in tables for each study: (i) author name and year of publication; (ii) study design, sample size, ICU population based on admission diagnosis (medical, surgical, etc.); (iii) IWS diagnosis criteria/tools; (iv) opioid and/or hypnotic reported in relation with IWS; (v) risk analysis; (vi) pharmacological and non-pharmacological strategies used for IWS treatment/prevention. Moreover, five study domains were defined and each manuscript fell into at least one depending on the following research questions: (1) “How is the diagnosis of IWS conducted in the adult ICU patient?”; (2) “How frequently is IWS reported in the adult ICU population?”; (3) “Which patients are at risk to develop IWS?”; (4) “What clinical effects and complications of IWS are described?” and (5) “What strategies were studied for IWS prevention and treatment?”

## 3 Results

**FIGURE 1:**
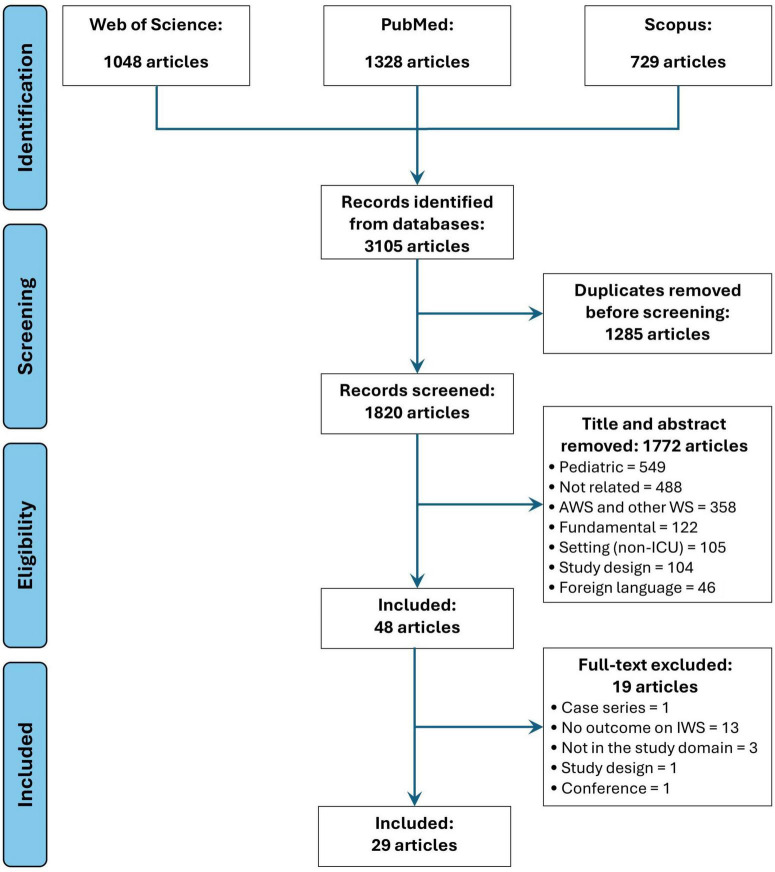
PRISMA flowchart diagram.

Article selection process for this scoping review is represented as a PRISMA Flow Diagram in Figure 1. Following the search strategy, 3,105 records were identified. A total of 29 original studies (7–13, 16–37) were selected for final analysis.

The topics covered are: (1) diagnosis, (2) epidemiology, (3) risk factors, (4) clinical effects and complications, (5) treatment and preventive strategies.

### 3.1 Diagnosis of IWS

Almost all studies included in the analysis [24 out of 29 (7–13, 16–37)] report criteria for IWS diagnosis. In none of the studies patients were exposed to only one drug known to induce withdrawal syndrome. However, to build our report in a structured manner, results will be presented based on the drug (opioids, hypnotics and α-2 agonists) primarily studied by the authors in relation to IWS. A summary of the main findings regarding IWS diagnosis are reported in Table 1 and Supplementary material 3.

**TABLE 1:** Comprehensive description of IWS diagnosis.

Criteria for IWS diagnosis	OR- and HR-IWS	α -2 agonists-related-IWS
DSM-5 criteria	(8, 10, 21, 23, 27, 28)	None
WAT-1 or COWS	(10, 27)	(20, 22)
Other diagnostic tools for withdrawal	(7, 11, 13)	None
Defines IWS arbitrarily	(12, 24, 25, 29)	(16, 17, 19, 20, 22, 26, 30, 31, 34, 35)
Specifies number of signs and symptoms required	(8, 10, 21, 23, 27, 28)	(17, 19, 20, 22, 30, 34, 35)
Defines specific signs and symptoms	(7, 8, 11, 13, 21, 23, 25, 28)	(16, 17, 19, 20, 22, 26, 30, 34, 35)
Differential diagnosis	(7–9, 21, 23, 27)	(16, 31, 34, 35)
Time of exposure	(7, 8, 10, 12, 21, 23–25, 27–29)	(16, 19, 20, 22, 26, 30, 35)
Drug regimen specified	(11, 24, 25, 28)	(16, 17, 19, 22, 26, 30, 31, 34, 35)
Defines drug weaning	(8, 11, 23–25, 27, 28)	(17, 19, 22, 30, 34, 35)
Concomitant use of other drugs	(7–13, 21, 23–25, 27–29)	(16, 17, 19, 20, 22, 26, 30, 34, 35)
Evaluation time	(8, 10, 11, 21, 23–25, 27–29)	(16, 17, 19, 20, 22, 26, 30, 31, 34, 35)
Exclusion criteria	(7, 8, 10–12, 21, 23–25, 27–29)	(16, 17, 19, 20, 22, 26, 30, 31, 34, 35)
IWS response to specific drugs	(7, 8, 11–13, 24, 25, 29)	(20, 22, 30, 35)

COWS, Clinical Opiate Withdrawal Scale; DSM-5, Diagnostic and Statistical Manual of Mental Disorders 5th edition; IWS, Iatrogenic Withdrawal Syndrome, HR, Hypnotic related; OR, Opioid Related; WAT-1, Withdrawal Assessment Tool.

#### 3.1.1 Opioid-related IWS (OR-IWS)

##### 3.1.1.1 Diagnosis of OR-IWS based on DSM-5 criteria

Opioid-related IWS diagnosis was defined in 14 studies (7–13, 21, 23–25, 27–29). In five prospective studies (8, 10, 21, 23, 27) and one retrospective report (28), OR-IWS was diagnosed based on the Diagnostic and Statistical Manual of Mental Disorders, 5th edition (DSM-5) criteria (38). The exposure time was arbitrarily defined as continuous or regularly intermittent for at least or more than 24 h (8, 10), 72 h (23, 27, 28), or 5 days (21). Opioid weaning and follow-up time for OR-IWS diagnosis varied among studies (8, 23, 27, 28). Moreover, hypnotics were used in conjunction with opioids in all patients (8, 10, 21, 23, 27, 28) but were weaned first only in 2 studies (8, 10). Furthermore, in 2 out of 6 studies, OR-IWS diagnosis and severity were compared against validated scales like Withdrawal Assessment Tool (WAT)-1 (27) and Clinical Opiate Withdrawal Scale (COWS) (10), respectively.

##### 3.1.1.2 Diagnosis of OR-IWS based on other scales

Other diagnostic tools were used in one retrospective (7) and 3 prospective studies (9, 11, 13). A minimum number of signs and symptoms (7, 9) or an intensity of OR-IWS (11, 13) was necessary to establish the diagnosis. In one retrospective study (7) including 28 surgical ICU patients, a composite set of withdrawal criteria were developed based on Himmelsbach’s scale (39) and other reports (40). These criteria were adjusted according to the need for clonidine during ICU stay (7). Two prospective reports assessed OR-IWS secondary to sufentanil (11) and remifentanil (13) using a 10-point scale by Jasinski (41). Neither study reported nor considered opioid exposure time for diagnosis (11, 13).

##### 3.1.1.3 Diagnosis of OR-IWS based on arbitrary criteria

In four studies [one retrospective (12), 1 prospective interventional (29) and 2 randomized control trials (RCT) (24, 25)], OR-IWS was defined based on arbitrary criteria comprising of signs and symptoms (12, 24, 25, 29), a specific time of exposure to opioids (12, 24, 29) and/or alleviation of OR-IWS to a certain drug (12). In a prospective study on 30 patients with IWS following a mixed opioid-hypnotic regimen until mechanical ventilation weaning, IWS was defined arbitrarily without detailed criteria (29).

#### 3.1.2 Hypnotic-related IWS (HR-IWS)

Six studies report IWS diagnosis on a mixed opioid-hypnotic regimen (7, 9, 11, 12, 21, 29). In five out of six studies, the authors did not use different diagnostic criteria for opioid and hypnotic withdrawal (7, 9, 11, 12, 29) and these were described in section 3.1.1. Only one prospective study specifically uses standardized diagnostic tools based on the DSM-5 and International Classification of Diseases Tenth Revision (ICD-10) criteria for benzodiazepine withdrawal (21). Iatrogenic withdrawal syndrome was arbitrarily defined and reported as a safety endpoint in three RCTs that compared various long-term sedation regimens (midazolam, dexmedetomidine, and propofol) (16, 17, 19). A rescue protocol for inadequate sedation was provided, but it is unclear whether it addressed IWS or tolerance development (16, 17, 19).

#### 3.1.3 α-2 agonists-related IWS (dexmedetomidine- and clonidine-related IWS (DR-IWS and CR-IWS)

The diagnosis of DR-IWS was reported in 8 studies (16, 17, 19, 20, 22, 26, 31, 35). Three studies directly investigated DR-IWS and at least two mandatory signs or symptoms were required to establish the diagnosis (20, 22, 35). Two reports used the WAT-1 score as one of the diagnostic criteria (20, 22). Other hypnotics and opioids were weaned either concurrently or before weaning dexmedetomidine (20, 22, 35). Five studies (one prospective single-arm (31), open-label phase III (26) and 3 RCTs (16, 17, 19)) reported DR-IWS as a safety endpoint. Few data could be extracted about CR-IWS (30, 34). Detailed results regarding DR-IWS and CR-IWS are reported in Table 1 and Supplementary material 3.

### 3.2 Epidemiology of IWS

Twenty-three studies with various designs (5 RCTs (16–19, 24), 12 prospective (8–10, 13, 20–23, 25–27, 30) and 6 retrospective (7, 12, 28, 31, 34, 35)) reported IWS incidence in critically ill adults (Table 2).

**TABLE 2:** Epidemiology of IWS.

Class	Agent	Incidence	Type of study	Authors	Year
Opioids	Sufentanil	149/466 (32%)	Single-center, prospective observational	Hofbauer et al. (25)	1999
	Remifentanil	80/587 (13.6%)	Single-center, prospective observational	Nseir et al. (13)	2009
	Fentanyl, methadone substitution	MG: 10/37 (27%)	Multicentric, double-blind randomized controlled trial	Wanzuita et al. (24)	2012
		CG: 12/31 (38.7%)			
	Any opioid	9/54 (16.7%)	2-center, prospective, observational	Wang et al. (23)	2017
	Any opioid	WAT-1: 19/52 (37%)	2-center, prospective, observational	Capilnean et al. (27)	2019
		DSM-5: 8/52 (15%)			
	Any opioid	37/126 (29.4%)	Single-center, retrospective cohort study	Hyun et al. (28)	2020
	Remifentanil	18/58 (31%)			
	Fentanyl	17/47 (36.2%)			
	Morphine	2/21 (9.5%)			
	Fentanyl	13/55 (23.6%)	Single-center, prospective observational	Taesotikul et al. (8)	2021
	Any opioid	COWS: 32/92 (35%)	Single-center, prospective observational	Fox et al. (10)	2023
		DSM-5: 27/92 (29%)			
Hypnotics	Midazolam	10/122 (8.2%)	Multicentric, double-blind, randomized controlled trial	Riker et al. (19)	2009
	Midazolam	8/250 (3.2%)	2 multicentric, double-blind, randomized controlled trials	Jakob et al. (16)	2012
	Propofol	7/247 (2.8%)	2 multicentric, double-blind, randomized controlled trials	Jakob et al. (16)	2012
	Propofol	36/208 (17%)	Multicentric, double-blind, randomized controlled trial	Hughes et al. (17)	2021
Opioid-hypnotic regimens		9/28 (32%)	Single-center, retrospective study	Cammarano et al. (7)	1998
		24/119 (20.1%)	Multicentric, prospective, observational	Sandiumenge et al. (9)	2016
		22/50 (44%)	Single-center, prospective observational	Arroyo-Novoa et al. (21)	2020
		57/115 (49.5%)	Single-center, retrospective cohort study	Maffei et al. (12)	2023
α-2 agonists	Dexmedetomidine	12/244 (4.9%)	Multicentric, double-blind, randomized controlled trial	Riker et al. (19)	2009
	Dexmedetomidine	MIDEX: 17/247 (6.9%)	2 multicentric, double-blind, randomized controlled trials	Jakob et al. (16)	2012
		PRODEX: 4/246 (1.6%)			
	Dexmedetomidine	2/75 (2.7%)	Multicentric, prospective, single-arm, phase III clinical trial	Ozaki et al. (26)	2014
	Dexmedetomidine	27/42 (64%)	Single-center, prospective observational	Bouajram et al. (20)	2019
	Dexmedetomidine	Clonidine group: 11/15—73%	Single-center, prospective, double cohort, pilot study	Bhatt et al. (22)	2020
		Control group: 16/27—59%			
	Dexmedetomidine	50/165 (30.3%)	2-center, retrospective study	Pathan et al. (35)	2021
	Dexmedetomidine	22/214 (10%)	Multicentric, double-blind, randomized controlled trial	Hughes et al. (17)	2021
	Clonidine	17.5%–69.5%	Single-center, retrospective cohort study	Purivatra et al. (34)	2021
	Dexmedetomidine	2/105 (2%)	Single-center, retrospective study	Fetters et al. (31)	2022

CG, control group; COWS, Clinical Opiate Withdrawal Scale; DSM-V, Diagnostic and Statistical Manual of Mental Disorders 5th Edition; IWS - Iatrogenic Withdrawal Syndrome; MG, methadone group; WAT-1, Withdrawal Assessment Tool-1.

Opioid- and opioid-hypnotic regimens-related IWS incidence was addressed in 7 observational studies (8, 10, 13, 23, 25, 27, 28) and one RCT (24), comprising a total of 1,693 patients with IWS rates ranging from 13.6 to 49.5%. When frequencies for different agents were reported, morphine had the lowest incidence of OR-IWS—9.5% (28). Two studies concurrently reported incidences based on DSM-5 criteria and a different diagnostic tool (WAT-1 and COWS (10, 27)), both alternatives yielding higher IWS rates.

Benzodiazepine-related IWS incidence was addressed as a safety endpoint in 2 RCTs reporting rates of 8.2% (10/122) and 3.2% (8/250), respectively (16, 19). Similarly, propofol-related IWS incidence was reported as safety endpoint in 2 RCTs: 17.3% (36/208) and 2.8% (7/247) respectively (16, 17).

α-2 agonists-related IWS incidence was investigated in 9 studies including 1,310 patients (16, 17, 19, 20, 26, 30, 31, 34, 35). Three multicentric RCTs addressed DR-IWS as safety endpoint and reported IWS rates between 4.9 and 10% (16, 17, 19). A higher incidence – 64%, was found in a cohort of patients with prolonged, i.e., over 72 h, infusion of dexmedetomidine (20). In clonidine-treated patients, a retrospective study found highly variable frequencies, 17.5–69.5%, depending on time since clonidine cessation and dose (34).

### 3.3 Risk factors for IWS

Twelve studies reported on potential risk factors associated with IWS in critically ill patients (7–10, 12, 20, 21, 23, 25, 32, 34, 35). For each drug class associated with IWS (opioid, opioid-hypnotic and α-2 agonists), identified risk factors fell into one of the following categories: patient-related, therapy-related or critical illness-related. A structured summary of factors independently associated with IWS is presented in Table 3 and further details are addressed below.

**TABLE 3:** Factors independently associated with IWS in multivariate analysis.

Class	Category	Risk factor	Multivariate analysis—Odds ratio (95% CI)
Opioid-hypnotic regimens	Patient-related	BMI	BMI ≥ 23 kg/m^2:^ 4.99 (1.42–17.60) (8)
		Alcohol abuse	5.3 (1.87–15.16) (9)
		Drug abuse	5.65 (1.82–17.51) (21)
	Therapy-related	Opioid cumulative dose	Fentanyl dose ≥ 1,200 μg/day—OR not reported (10) 1.14 (1.05–1.23) (21)
		Opioid infusion duration	Infusion duration ≥ 72 h—OR not reported (10) 0.70 (0.50–0.97) (21); 1.08 (1.02–1.14) (12)
		Weaning rate ≥ 50 μg fentanyl/h	9.66 (1.51–61.92) (8)
		Concomitant benzodiazepine use	3.02 (1.12–8.15) (12) (for lorazepam)
		Number of concurrent sedatives	4.9 (CI 1.85–13.28) (9)
	Critical illness-related	RASS score	4.13 (2.09–8.16) (21)
		Delirium	2.69 (1.01–7.14) (21)
α-2 agonists	Therapy-related	Dexmedetomidine cumulative dose	Dose greater than 12.9 μg/kg/d: 4.9 (1.2–20.3) (20)
		Dexmedetomidine peak rate	Rates over 0.8 μg/kg/hr: 8 (1.8–35.7) (20)

BMI, Body Mass Index; CI, Confidence Interval; OR, Odds Ratio; RASS, Richmond Agitation Sedation Scale.

#### 3.3.1 Patient-related risk factors in patients with OR- and HR-IWS

Opioid- and opioid-hypnotic regimens-related risk factors for IWS were assessed in 6 prospective (8–10, 21, 23, 25) and 2 retrospective (7, 12) studies including 979 patients. As for patient-related factors, one of the earliest studies reported that IWS-positive patients were significantly younger (7), but no other study replicated this finding later on (8, 10, 12, 21, 23, 25). Patients with a BMI ≥ 23 kg/m^2^ had 5-times higher odds of developing IWS (8). Heavy alcohol consumption (9) and a history of drug use (21) also increased the risk of developing IWS up to 500%. Other studies did not confirm this finding, likely due to a reduced number of patients with concurrent substance abuse included in their cohorts (7, 12).

#### 3.3.2 Therapy-related risk factors in patients with OR- and HR-IWS

In terms of therapy-related risk factors, a substantial number of studies reported higher opioid doses (7, 10, 12, 21), opioid infusion duration (10, 12, 21) and increased opioid weaning rates (8) in IWS-positive patients. Potential clinically valuable thresholds were reported for fentanyl: infusion duration over 72 h (10), dose > 1,200 μg/day (10) and a weaning rate > 50 μg/h (8). Concomitant use and higher doses of sedatives, such as benzodiazepines (7, 10, 12, 21), propofol (7, 12) and dexmedetomidine (12) are also more prevalent in IWS-positive groups. No definitive conclusion can be drawn concerning the effect of antipsychotics (7, 10, 23) and neuromuscular blocking agents use (7, 10, 12) due to conflicting results. Naloxegol use to prevent constipation in critically ill adults receiving parenteral opioids did not influence COWS scores in a double-blind RCT (32).

#### 3.3.3 Critical illness-related risk factors in OR- and HR-IWS

Critical illness-related risk factors such as Acute Respiratory Distress Syndrome (ARDS) diagnosis (7), higher Richmond Agitation Sedation Scale (RASS) score and delirium incidence (21) were observed in IWS-positive subgroups.

#### 3.3.4 Risk factors for α-2 agonists-related IWS

α-2 agonists-related IWS risk factors were investigated in 3 studies (2 retrospective and 1 prospective) on 373 patients (20, 34, 35). Among therapy-related risk factors, peak dexmedetomidine rates over 0.8 μg/kg/h and cumulative daily doses greater than 12.9 μg/kg were independently linked to IWS in multivariable analysis (20). Other factors regarding dexmedetomidine posology and kinetics did not affect withdrawal rates (20, 35). Higher rates of concurrent opioid and benzodiazepine discontinuation were observed in IWS-positive groups (35). Clonidine dosing regimens also did not influence symptoms (34).

### 3.4 Clinical effects and complications of IWS

Twelve studies reported clinical effects related to IWS development during ICU stay, pertaining to one of the following categories: hospitalization- and ICU-related outcomes (8, 11–13, 20, 21, 23, 35–37), physiological effects (11, 12, 20, 29, 35) and long-term outcomes (33). These will be presented below for opioid and/or hypnotic regimens and α-2 agonists.

#### 3.4.1 Hospitalization- and ICU-related outcomes in OR- and HR-IWS

Hospitalization- and ICU-related outcomes were reported in 8 studies on 992 patients (8, 11–13, 21, 23, 36, 37). Results regarding the duration of mechanical ventilation are conflicting. Three studies (2 prospective, 1 retrospective) found prolonged periods of mechanical ventilation in IWS positive patients (12, 21, 23), while one prospective study did not confirm this finding (8). Similarly, no definitive conclusion could be inferred concerning the impact of IWS on ICU and hospital LOS (8, 23). One prospective study on 587 patients found a 2.6 times higher risk of ICU-acquired infections in patients experiencing remifentanil IWS (13). However, this finding could not be replicated by 2 smaller studies on post-surgical critically ill patients after remifentanil cessation (36, 37). Most of these results are conflicting and no definitive conclusion can be made. Prospective studies with large cohorts and adequate design, as well as a meta-analysis or a sensitivity analysis could resolve this issue, but this is beyond the scope of a scoping review.

#### 3.4.2 Physiological effects of OR- and HR-IWS

Physiological effects were described in 3 prospective studies (11, 12, 29). One study on 29 patients treated with sufentanil and midazolam or propofol, respectively, registered hemodynamic variations in both groups following weaning and found no association with withdrawal intensities (11). Another prospective study on 30 patients with failed mechanical ventilation weaning due to withdrawal symptoms reported significant increases of hemodynamic parameters, minute ventilation, resting energy expenditure (REE), oxygen consumption (V_*O*2_) and carbon dioxide production (V_*CO*2_) (29). One study on ARDS patients evaluated P/F ratios at 7 and 14 days and found a tendency toward better oxygenation indices at 14 days in IWS-positive group (12); nevertheless, a survival bias could have interfered with the results. Neurophysiological parameters such as β-endorphin, met-enkephalin levels and the amplitude height of the somato-sensory evoked potentials (SSEP) after weaning correlated with withdrawal intensity for both regimens (11).

#### 3.4.3 Long-term outcomes in OR- and HR-IWS

Long-term outcomes have been investigated in an exploratory study on trauma ICU survivors who had participated in a previous study on opioid and benzodiazepine withdrawal (33). Out of the 5 patients with chronic opioid use after discharge which reported withdrawal symptoms at home within the first 4 months, 2 had also experienced probable withdrawal during ICU stay (33).

#### 3.4.4 Clinical effects and outcomes in α-2 agonist-related IWS

Complications regarding α-2 agonist-related IWS were not directly addressed in any selected study. No difference was observed between patients with DR-IWS compared to those without DR-IWS regarding ICU LOS (20, 35). Although some adverse events (hemodynamic alterations, delirium, agitation, nausea and vomiting, etc.) could be interpreted as complications of DR-IWS, all of these were taken into consideration as diagnosis criteria for DR-IWS (20, 35).

### 3.5 Treatment and preventive strategies of the IWS

A few clinical studies (22, 24, 29–31, 42) directly investigated the efficacy of therapeutic or prophylactic interventions for IWS in ICU patients. Nevertheless, many investigators arbitrarily used antipsychotics, sedatives or re-administration of the causative agents to alleviate or prevent withdrawal symptoms (7, 8, 11–13, 20, 25, 35).

One double-blind RCT evaluated the effect of enteral methadone during weaning from sedation and analgesia on 68 mechanically ventilated critically ill patients (24). Despite non-significant differences in withdrawal symptoms frequency, methadone group had a higher probability of a quicker, successful extubation (24).

Another prospective study showed the efficacy of intravenous clonidine in restoring hemodynamic, respiratory and metabolic (REE, V_*O*2_ and V_*CO*2_) parameters back to baseline levels in patients with IWS (29). Moreover, non-responders had longer mechanical ventilation duration, while most responders were extubated within 2 days (29).

Three studies reported bridging strategies with enteral clonidine for prolonged dexmedetomidine infusions (22, 30, 42) and all of them concluded that this strategy is safe and feasible. No difference in the incidence of withdrawal symptoms or WAT-1 scores was found between a clonidine taper and weaning off dexmedetomidine alone, but patients receiving clonidine had better RASS scores (22). Lastly, one retrospective study reported the use of enteral guanfacine for dexmedetomidine cessation in 105 ICU patients (31). Weaning was achieved in 58% of patients and the frequency of DR-IWS was 2% (31). In the absence of a control group, no conclusion can be draw regarding guanfacine efficacy in preventing DR-IWS (31).

## 4 Discussion

The present scoping review is the first to report on diagnosis, epidemiology, risk factors, complications, clinical effects, treatment and preventive strategies of IWS. To date, no consensus from the critical care societies has been made regarding IWS definition and management. In this manuscript we mapped the available data, and we demonstrated the lack of consistency among clinical reports regarding IWS diagnosis (7–13, 16, 17, 19–29, 31, 34, 35). In our opinion, this is the most important aspect reported since a heterogenous definition of IWS will: (i) directly affect the incidence, (ii) alter the methods of studies focusing on complications and predictive models; (iii) be a major limitation in RCTs aiming to prevent or treat IWS. These observations will be further discussed.

The heterogeneity of diagnosis criteria highlighted in the results section is marked by a chronological trend in IWS definition. Studies between 1998 and 2016 (7, 9, 11, 13, 23–25, 29) diagnosed OR- and HR-IWS based mostly on arbitrary criteria, or scales (39–41). The criteria outlined have not been replicated in studies published since 2017, where the DSM-5 criteria were predominantly applied (8, 10, 21, 23, 27, 28). Considering the critical issues surrounding IWS diagnosis, it is imperative for the scientific community to work toward establishing a new, unified definition of IWS in ICU patients. In our opinion, this definition should be developed based on a few principles. Firstly, it should follow the DSM-5 framework (38), due to its wide scientific acceptance as a fundamental definition of withdrawal. Also, a common pathway for OR-IWS and HR-IWS diagnosis should be designed given their widespread concomitant use in ICU patients. We consider that a distinct pathway should address the IWS related to α-2 agonists. Secondly, studies published since 2017 (8, 10, 21, 23, 27, 28) may serve as a sounder scientific basis, considering that these show greater coherence, more rigorous methodology and are mainly based on DSM-5 criteria. Lastly, given the complexity in IWS diagnosis resulting from current studies, a clear and easy to use definition should be an adaptation from the DSM-5 criteria in the context of critical care medicine. Taking into consideration these principles, we propose that a new IWS definition should encompass three diagnostic domains: (i) a refined definition of the terms *heavy and prolonged use*, as well as *abrupt cessation*; (ii) the presence of at least 3 signs and symptoms associated with withdrawal either to opioids or hypnotics (including α-2 agonists) as per DSM-5, not due to other critical illnesses and developing within a defined follow-up period after drug cessation; (iii) signs and symptoms are precipitated or alleviated following the administration of an antagonist or an agonist to the inciting drug, respectively. On a final note, a systematic review on diagnostic and management strategies of IWS in pediatric and adult ICU which analyzed studied published between 1946 and 2017 concluded that in adult patients, no validated tool for IWS diagnosis could be identified (43).

With regard to risk factors reporting, a significant concern is that only a limited number of studies conducted a valid statistical analysis to ascertain an independent relationship with IWS (8–10, 12, 20, 21). Upon reviewing the studies that employed multivariate logistic regression analysis, it is noteworthy that none of them IWS (8–10, 12, 20, 21) adhered to the standards set by a scientifically approved checklist, such as the Transparent Reporting of a multivariable prediction model for Individual Prognosis Or Diagnosis (TRIPOD) (44). In terms of predictive model development, only two authors reported the full regression models (12, 21), while none used a Cox regression model. Three authors (12, 20, 21) provided a clear rationale or method for the variables introduced in the models, while in all the other studies, this aspect, as well as the exact number of variables introduced, is less clear (8–10). Lastly, one study indicated a higher risk of multicollinearity by introducing possibly strongly correlated variables in the same models (21). We consider that future studies should conduct a robust statistical analysis, including the following aspects: (i) the full regression model; (ii) rationale and method of variable selection; (iii) model’s goodness-of-fit and avoidance of multicollinearity; (iv) appropriate number of variables per studied event frequency; (v) clinically sound adjustment of the studied factors; (vi) effect of variables upon the time of event. Despite these important issues that must be addressed, a few observations from these studies are noteworthy. The risk of IWS development was strongly related to drug prescription (dose, duration, weaning) strategies (7, 8, 10, 20, 21) and the number of drug classes used (8–10, 12, 23, 32, 34, 35). Chronic alcohol and drug use may serve as a significant risk factor and confounder, thereby complicating differential diagnosis (9, 21), given that most studies have excluded these patients (7, 10, 11, 17, 21–23, 26, 27, 31, 35). Patient-, critical illness- and institutional-related risk factors must be analyzed in future high-quality studies.

Preventive strategies could be derived from factors previously associated with IWS (7–10, 12, 20, 21, 23, 35). Key strategies may include reducing infusion duration, administering lower doses of opioids and/or hypnotics, selecting drugs with optimal kinetic and dynamic profiles, avoiding benzodiazepines, and implementing careful weaning protocols. A risk score or scale with relevant discriminative power could be developed in future studies and subsequently implemented through best practice guidelines. Current evidence shows that most ICUs rarely have a weaning or IWS protocol and out of the few ICUs in which such protocols exist, these are hardly implemented at bedside (5, 45). Also, the paucity of standardized knowledge on IWS is stressed by all professionals in the field (6, 46). One qualitative study found that most critical care nurses did not receive any education or training on IWS assessment and management (6). Critical care nurses noted a general lack of awareness and knowledge regarding IWS among all ICU professionals, emphasizing that this condition is often overlooked by both doctors and nurses. Consequently, adequate therapeutic measures are not considered in a patient experiencing IWS. Lastly, nurses suggested that these issues can be corrected through proper training, implementation of validated assessment tools and better communication with the attending physicians and other members from the ICU team (6).

Four studies, including one RCT (24), one interventional (29), one prospective (22), and one retrospective observational (31) study focused on opioid or α-2 agonists weaning strategies mainly including substitution therapy. Although no study observed a significant difference in IWS rates, some of them found a shorter time to extubation (24, 29). Additionally, apart from one study (29), none of the previously mentioned interventions directly address IWS treatment (22, 31). These only report the frequency of IWS signs and symptoms following the cessation of the inciting drug. Two studies (47, 48) not included in our analysis indicate a shorter period for mechanical ventilation (48) and faster opioid infusion weaning when methadone substitution therapy is initiated (47). These studies did not report any data on IWS. The lack of robust data on IWS treatment and prevention is an issue of utter importance stressed in other systematic review conducted on neonate, pediatric and adult patients with IWS (49). High-quality randomized clinical trials must be conducted in the future regarding treatment and prevention, but several methodological issues arise. Firstly, the lack of a unified definition will lead to a recruitment bias. Secondly, effective tapering protocols are challenging to implement given that a mixed opioid-hypnotic regimen is widely used, and previous studies must serve as a starting point. Thirdly, given that risk factors are not completely described, adjustment of interventions will be limited. Lastly, the safety of these interventions will be limited by the incomplete description of IWS clinical effects on organ systems.

While we summarized significant evidence in the present scoping review, there are limitations. Firstly, many studies were conducted on small cohorts, from different ICU settings and with potential geographical bias. Thus, these findings should not be generalized to all populations. Secondly, we also gathered data from papers reporting IWS as secondary or safety end point, limiting our ability to highlight major methodological features. Furthermore, we were unable to address in a comprehensive manner issues regarding mechanisms, prevention and treatment of IWS, mostly due to a low body of data or ambiguous management of IWS. Lastly, although widely used, relevant literature about α-2 agonists related-IWS was scarce.

## 5 Conclusion

In conclusion, we consider IWS to be a prevalent entity among adult critically ill patients, despite being inconsistently defined and approached. This scoping review mapped significant evidence related to IWS and identified key knowledge gaps, providing possible directions for future studies. Until high-quality data is available, awareness should be raised on IWS during our daily practice.
