# Supplementary material for: Iatrogenic withdrawal syndrome in adult intensive care unit: a scoping review
Source: Front Med (Lausanne). 2025 Jul 23;12:1573363. doi: 10.3389/fmed.2025.1573363 (PMC12325356; doi:10.3389/fmed.2025.1573363)
Supplement: Supplementary file 2 [file Table_2.docx]

Supplementary Material 2

**Methods - Search strategy**

1. **Search string depending on database:**
2. **Search string for PubMed: 1328 manuscripts (01.01.1990-30.09.2024)**

(opioid OR benzodiazepine OR ketamine OR hypnotic OR propofol OR sevoflurane OR clonidine OR dexmedetomidine OR barbiturate) AND (withdrawal OR withdrawal syndrome OR iatrogenic withdrawal OR iatrogenic withdrawal syndrome) AND (intensive care OR ICU OR intensive care unit OR critical care)

1. **Search string for Scopus: 729 manuscripts (01.01.1990-30.09.2024)**

TITLE-ABS (benzodiazepine OR opioid OR hypnotic OR ketamine OR dexmedetomidine OR propofol OR sevoflurane OR clonidine OR barbiturate ) AND TITLE-ABS ( withdrawal OR withdrawal-syndrome OR iatrogenic-withdrawal ) AND TITLE-ABS ( intensive-care OR icu OR critical-care ) AND PUBYEAR > 1989 AND PUBYEAR < 2025 AND PUBYEAR > 1989 AND PUBYEAR < 2025 AND PUBYEAR > 1989 AND PUBYEAR < 2025

1. **Search string for Web of Science: 1048 manuscripts (01.01.1990-30.09.2024)**

ALL=((opioid OR hypnotic OR benzodiazepine OR propofol OR ketamine OR dexmedetomidine OR clonidine OR sevoflurane OR barbiturate) AND (withdrawal OR withdrawal syndrome OR iatrogenic withdrawal) AND (intensive care OR ICU OR critical care)
