# Supplementary material for: Iatrogenic withdrawal syndrome in adult intensive care unit: a scoping review
Source: Front Med (Lausanne). 2025 Jul 23;12:1573363. doi: 10.3389/fmed.2025.1573363 (PMC12325356; doi:10.3389/fmed.2025.1573363)
Supplement: Supplementary file 3 [file Table_3.docx]

Supplementary Material 3

**Table S1. Comprehensive description of IWS diagnosis**

|  | **Author, year and type of study** | **Drug used and IWS incidence** | **Diagnostic criteria** | **Structured description of IWS diagnosis** |
| --- | --- | --- | --- | --- |
| 1 | Cammarano et al. [1]  1998  Retrospective  Mixed ICU cohort | Opioids  Benzodiazepine, Propofol  (equivalents of fentanyl and lorazepam)  Epidural and oral opioids were taken into consideration and conversion of potency compared with intravenous route was made  **IWS incidence:** 9/28 (32%) | **IWS as a primary outcome**  A composite set of withdrawal criteria was made based on Himmelsbach’s scale [2]and other criteria [3] (2 signs and 2 symptoms).  The number of criteria was modified according to the need for clonidine during LOS or if there was a record of AWS during LOS | **No. of signs and symptoms required:** yes, as per composite scale  **Time of exposure:** >7 days  **Defines specific signs and symptoms:** yes  **Differential diagnosis:** yes  **Opioid regimen specified:** no  **Concomitant hypnotic use**: yes  **Defines opioid weaning:** not specified  **Evaluation time:** not specified  **Exclusion criteria:** yes  **IWS response to specific drugs:** yes  **Sedatives weaned first:** not specified |
| 2 | Hofbauer et al. [4]  1999  Prospective, observational, single centre  Medical ICU patients under MV | Primarily focusing on sufentanil  Midazolam as a background infusion  **IWS Incidence:** 149/466 (32%) | Arbitrarily defined as presence of agitation, sweating, shivering.  **IWS as a secondary outcome** | **No. of signs and symptoms required:** not specified  **Time of exposure:** ≥96 hours  **Defines specific signs and symptoms:** yes  **Differential diagnosis:** not specified  **Opioid regimen specified:** yes  **Concomitant hypnotic use**: yes  **Defines opioid weaning regimen:** yes  **Evaluation time:** twice daily since the start of opioid weaning  **Exclusion criteria:** yes  **IWS response to specific drugs:** yes  **Sedatives weaned first:** yes |
| 3 | Korak-Leiter et al. [5]  2005  Prospective  Surgical ICU patients under MV | Patients were randomly allocated to receive either a midazolam based or a propofol-based sufentanil sedation regimen  Sufentanil-Propofol vs. Sufentanil-Midazolam  **IWS Incidence:** not reported | Criteria for IWS presence and intensity as defined by Jasinski et al. [6]. A scoring system taking 10 signs and symptoms into account.  **IWS as a primary outcome following two different opioid-hypnotic regimens** | **No. of signs and symptoms required:** IWS presence and intensity as defined by Jasinski et al. [6]  **Time of exposure:** not specified  **Defines specific signs and symptoms:** yes  **Differential diagnosis:** no  **Opioid regimen specified:** yes  **Concomitant hypnotic use**: yes  **Defines opioid weaning regimen:** yes  **Evaluation time:** 2 independent clinicians not involved in the study; from weaning initiation up to 24 hours at 6 h intervals  **Exclusion criteria:** yes  **IWS response to specific drugs:** yes  **Sedatives weaned first:** concomitant with opioids |
| 4 | Liatsi et al. [7]  2009    Prospective, intervenional, single centre  Trauma patients under MV | Remifentanil-propofol (first 24h)  Fentanyl-midazolam (following days)  **IWS Incidence:** only patients with IWS were analyzed | Defined arbitrarily  Signs and symptoms: profound sweating, agitation, hyperventilation, hypertension and tachycardia.  **Primary outcome: effect of intravenous clonidine on hemodynamic, respiratory and metabolic effects of IWS** | **No. of signs and symptoms required:** not specified  **Time of exposure:** ≥24 hours  **Defines specific signs and symptoms:** no  **Differential diagnosis:** no  **Opioid regimen specified:** no  **Concomitant hypnotic use**: yes  **Defines opioid weaning regimen:** no  **Evaluation time:** all patients developed IWS in the 10-20 min recording period; clonidine was administered to all patients  **Exclusion criteria:** only patients under MV were evaluated  **IWS response to specific drugs:** yes (clonidine)  **Sedatives weaned first:** concomitant weaning of opioids and hypnotics |
| 5 | Nseir et al. [8]  2009  Prospective, observational, single center  Mixed ICU | Remifentanil  **IWS Incidence:** 80/587 (13.6%) | Same methodology as Korak-Leiter et al. [5] regarding IWS definition  **IWS related to the primary outcome** | **No. of signs and symptoms required:** as per Korak-Leiter et al  **Time of exposure:** not specified  **Defines specific signs and symptoms:** yes  **Differential diagnosis:** not specified  **Opioid regimen specified:** partially  **Concomitant hypnotic use**: yes  **Defines opioid weaning:** no  **Evaluation time:** no  **Exclusion criteria:** no  **IWS response to specific drugs:** yes  **Sedatives weaned first:** mixed |
| 6 | Riker et al. [9]  2009  Prospective, multicentric, double-blind RCT  Mixed ICU | Midazolam  Dexmedetomidine  **IWS Incidence:**  Midazolam: 10/122 (8.2%)  Dexmedetomidine: 12/244 (4.9%) | Defined as at least 1 event in 24h after drug cessation, consisting of: agitation, headache, hyperhidrosis, nausea, nervousness, tremor, or vomiting)  Authors used the same criteria for both drugs.  **IWS as a secondary outcome (safety endpoint)** | **No. of signs and symptoms required:** ≥1  **Time of exposure:** ≥72 hours  **Defines specific signs and symptoms:** yes  **Differential diagnosis:** not specified  **α-2 agonist/hypnotic regimen specified:** yes  **Concomitant opioid use**: yes, fentanyl in boluses, only when needed  **Defines α-2 agonist/midazolam weaning regimen:** yes  **Evaluation time:** yes, 48 hours  **Exclusion criteria:** yes  **IWS response to specific drugs:** not clearly specified **(rescue)**  **Sedatives/opioids weaned first:** not specified |
| 7 | Wanzuita et al. [10]  2012  Double-blind, multicentric RCT  Mixed ICU patients under MV | Opioids (fentanyl)  **IWS Incidence:** MG: 27% (10/37)  **IWS Incidence:** CG: 38.7% (12/31) | Occasional episodes of intolerance to opioid withdrawal, characterized by agitation, anxiety, tremors, myoclonus, vomiting, diarrhea, piloerection, sweating, dilated pupils, tachycardia and hypertension. Midazolam was used as a main hypnotic together with opioids.  **IWS as a secondary outcome** | **No. of signs and symptoms required:** not specified  **Time of exposure:** 2 definitions (≥5 days of fentanyl in varying doses or ≥5 μg/kg/h for ≥12 hours)  **Defines specific signs and symptoms:** no  **Differential diagnosis:** not specified  **Opioid regimen specified:** yes  **Concomitant hypnotic use**: yes  **Defines opioid weaning:** yes  **Evaluation time:** yes  **Exclusion criteria:** yes  **IWS response to specific drugs:** yes  **Sedatives weaned first:** not clearly specified |
| 8 | Jakob et al. [11]  2012  Two double-blind, multicentric RCTs  Mixed ICU patients under MV | Propofol-Dexmedetomidine  **IWS Incidence:**  Propofol: 7/247 (2.8%)  Dexmedetomidine: 4/246 (1.6%)  Midazolam-Dexmedetomidine  **IWS Incidence:**  Midazolam: 8/250 (3.2%)  Dexmedetomidine: 17/247 (6.9%) | IWS defined as signs of excessive sympathetic activity (such as agitation, sweating, tremor, palpitation, anxiety, nausea and headache). The investigator decided if these should be interpreted as IWS or to be taken separately.  Authors used the same criteria for all drugs.  **IWS as a secondary outcome (safety endpoint)** | **No. of signs and symptoms required:** no  **Time of exposure:** ≥24 hours  **Defines specific signs and symptoms:** yes  **Differential diagnosis:** yes  **α-2 agonist/hypnotic regimen specified:** yes  **Concomitant opioid use**: yes  **Defines α-2 agonist/propofol weaning regimen:** no  **Evaluation time:** 48 hours  **Exclusion criteria:** yes  **IWS response to specific drugs:** not specified (rescue drugs)  **Sedatives/opioids weaned first:** not specified |
| 9 | Ozaki et al. [12]  2014  Prospective, single-arm, open-label, multicenter, phase III study  Mixed ICU | Dexmedetomidine  **IWS Incidence:** 2/75 (2.7%) | Secondary safety assessments as withdrawal symptom-related adverse events (including increased blood pressure, tachycardia, nausea/vomiting, headache, tremor, anxiety, sweating, or agitation), and rebound assessments of the post-infusion changes in mean arterial blood pressure, HR, and rate-pressure product.  **IWS as a secondary outcome (safety endpoint) in long-term sedation with dexmedetomidine** | **No. of signs and symptoms required:** not specified  **Time of exposure:** >24 hours  **Defines specific signs and symptoms:** yes  **Differential diagnosis:** no  **α-2 agonist regimen specified:** yes  **Concomitant hypnotic/opioid use**: yes  **Defines α-2 agonist weaning regimen:** no  **Evaluation time:** yes  **Exclusion criteria:** yes  **IWS response to specific drugs:** not specified  **Sedatives/opioids weaned first:** mixed |
| 10 | Gagnon et al. [13]  2015  Prospective, observational, pilot study  Mixed ICU | Clonidine  **IWS Incidence:** 1/20 (5%) | **Objective symptoms:** sustained hyper  tension (BP > 180/120 mm Hg) or tachycardia (HR > 120 beats/min) representing a change from baseline.  **Subjective symptoms** included sustained headache,  nervousness, insomnia, palpitations, anxiety,  restlessness, tremor, emotional instability, flushing, or vomiting.  IWS as a secondary outcome (safety data) | **No. of signs and symptoms required:** ≥1  **Time of exposure:** ≥24 hours  **Defines specific signs and symptoms:** yes  **Differential diagnosis:** no  **α-2 agonist regimen specified:** yes  **Concomitant hypnotic/opioid use**: yes  **Defines α-2 agonist weaning regimen:** yes  **Evaluation time:** yes, 48 hours  **Exclusion criteria:** yes  **IWS response to specific drugs:** yes  **Sedatives/opioids weaned first:** mixed |
| 11 | Sandiumenge et al. [14]  2016  Prospective, observational, multicentric,  Mixed ICU patients under MV | Sedative-analgesic regimens including midazolam, propofol, fentanyl, morphine, remifentanil  **IWS Incidence:** 24/119 (20.1%) | **Defined arbitrarily by national guidelines**  **Primary outcome:** impact of harmful use of alcohol on the sedation of patients under MV  Sedation withdrawal studied as a complication  **IWS as a secondary outcome** | **No. of signs and symptoms required:** as defined by national guidelines  **Time of exposure:** defines short (<72 hours) and prolonged (≥72 hours) exposure to sedatives and analgesics, **but not for IWS**  **Defines specific signs and symptoms:** yes  **Differential diagnosis:** yes  **Hypnotic regimen specified:** no  **Concomitant opioid use**: yes  **Defines hypnotic/opioid weaning regimen:** no  **Evaluation time:** no  **Exclusion criteria:** no  **IWS response to specific drugs:** not specified  **Sedatives/opioids weaned first:** not specified |
| 12 | Wang et al. [15]  2017  Prospective, observational, multicenter  Trauma ICU patients under MV | Opioids  Benzodiazepines  (converted to fentanyl and midazolam equivalents)  **IWS Incidence:** 9/54 (16.7%) | **Primary outcome:** incidence of IWS in MV adult ICU patients and possible risk factors  No regression analysis for risk factors | **No. of signs and symptoms required:** as per DSM-5  **Time of exposure:** ≥72 hours  **Defines specific signs and symptoms:** yes  **Differential diagnosis:** yes  **Opioid regimen specified:** not specified  **Concomitant hypnotic use**: yes  **Defines opioid weaning:** yes  **Evaluation time:** yes  **Exclusion criteria:** yes  **IWS response to specific drugs:** no  **Sedatives weaned first:** not specified |
| 13 | Bouajram et al. [16]  2019  Prospective, observational, single center  Mixed ICU | Dexmedetomidine  **IWS Incidence:** 27/42 (64%) | **Primary outcome:** incidence of dexmedetomidine withdrawal in adult ICU patients and risk factors for dexmedetomidine induced-IWS  Criteria used: tachycardia, hypertension, RASS > +1, positive CAM-ICU, WAT-1 score ≥ 3. | **No. of signs and symptoms required:** ≥2 during a single assessment  **Time of exposure:** >72 hours  **Defines specific signs and symptoms:** yes  **Differential diagnosis:** not specified  **α-2 agonist regimen specified: no**  **Concomitant hypnotic/opioid use**: yes  **Defines α-2 agonist weaning regimen:** no  **Evaluation time:** yes, from decrease in peak rate/when doses reduced with the aim of weaning up to 48 hours after discontinuation  **Exclusion criteria:** yes  **IWS response to specific drugs:** yes  **Sedatives/opioids weaned first:** not specified |
| 14 | Capilnean et al. [17]  2019  Prospective, bicentric, pilot study,  Trauma ICU patients under MV | Opioids  **IWS Incidence:**   \| 19/52 (37%) \| WAT-1 \| \| --- \| --- \| \| 8/52 (15%) \| DSM-V \| | DSM-5 and WAT-1  2 groups: pharmacists evaluated with WAT-1, intensivists with DSM-5  Groups blinded to the results  Interrater agreement, sensitivity and specificity of WAT-1 to DSM-5 evaluated  **Primary outcome: validation of WAT-1 against DSM-5 criteria for IWS in adult ICU** | **No. of signs and symptoms required:** as per DSM-V and WAT-1  **Time of exposure:** ≥72 hours  **Defines specific signs and symptoms:** no  **Differential diagnosis:** yes  **Opioid regimen specified:** no  **Concomitant hypnotic use**: yes  **Defines opioid weaning regimen:** yes  **Evaluation time:** at opioid weaning initiation, twice-daily on weekdays, once-daily on week-end; gives specific follow-up times  **Exclusion criteria:** yes  **IWS response to specific drugs:** not specified  **Sedatives weaned first:** not specified |
| 15 | Bhatt et al. [18]  2020  Prospective, single center, double cohort  Mixed ICU | Dexmedetomidine  **IWS Incidence:**  Clonidine group: 11/15 – 73%  Control group: 16/27 – 59% | **Primary outcome:**  to compare the incidence of dexmedetomidine withdrawal symptoms in ICU patients transitioning to a clonidine taper versus those weaned off dexmedetomidine alone after at least 3 days of continuous infusion  Five withdrawal symptoms evaluated were as follows: (1) agitation (RASS > +1), (2) delirium (positive CAM-ICU), (3) (WAT-1) score > 2, (4) tachycardia (>90 bpm), and (5) hypertension (SBP > 140 mm Hg or MAP > 90 mm Hg) | **No. of signs and symptoms required:** ≥2 during a single assessment within 24 hours after dexmedetomidine cessation  **Time of exposure:** ≥72 hours  **Defines specific signs and symptoms:** yes  **Differential diagnosis:** not specified  **α-2 agonist regimen specified:** yes  **Concomitant hypnotic/opioid use**: yes  **Defines α-2 agonist weaning regimen:** yes  **Evaluation time:** yes, up to 24 hours  **Exclusion criteria:** yes  **IWS response to specific drugs: yes**  **Sedatives/opioids weaned first:** not specified |
| 16 | Arroyo-Novoa et al. [19]  2020  Prospective  Trauma ICU | Opioids  Benzodiazepine  (morphine and lorazepam equivalents)  **IWS Incidence:** 22/50 (44%) | Developed a checklist based on DSM-5, ICD-10 and previous research.  Based on the number of signs and symptoms patients could fall in one of the following categories: probable, no and questionable.  **IWS as a primary outcome**  . | **No. of signs and symptoms required:** yes  **Time of exposure:** ≥5 days  **Defines specific signs and symptoms:** yes  **Differential diagnosis:** yes  **Opioid regimen specified:** no  **Concomitant hypnotic use**: yes  **Defines opioid weaning:** no  **Evaluation time:** yes, twice daily up to 72 hours since weaning started  **Exclusion criteria:** yes  **IWS response to specific drugs:** not specified  **Sedatives weaned first:** not specified |
| 17 | Hyun et al. [20]  2020  Retrospective  Medical ICU patients under MV for >72 hours | Opioids  **IWS Incidence:**   \| Remifentanil \| 18/58 (31%) \| \| --- \| --- \| \| Fentanyl \| 17/47 (36.2%) \| \| Morphine \| 2/21 (9.5%) \| | DSM-5 and several pediatric tools (not specified which tools)  **IWS as a primary outcome** | **No. of signs and symptoms required:** ≥3 for >2 hours simultaneously  **Time of exposure:** >72 hours  **Defines specific signs and symptoms:** 5 CNS and 6 ANS  **Differential diagnosis:** no  **Opioid regimen specified:** yes  **Concomitant hypnotic use**: yes  **Defines opioid weaning regimen:** yes  **Evaluation time:** up to 24 hours after opioid cessation  **Exclusion criteria:** yes  **IWS response to specific drugs:** not specified  **Sedatives weaned first:** not specified |
| 18 | Taesotikul et al. [21]  2021  Prospective, observational, single center  Mixed ICU patients under MV | Opioids  **IWS Incidence:**  13/55 (23.6%) | DSM-5 criteria  **IWS as a primary outcome** | **No. of signs and symptoms required:** as per DSM-5  **Time of exposure:** ≥24 hours  **Defines specific signs and symptoms:** yes  **Differential diagnosis:** yes  **Opioid regimen specified:** no  **Concomitant hypnotic use**: yes  **Defines opioid weaning:** yes  **Evaluation time:** yes, six time-points from 0 to 72 hours  **Exclusion criteria:** yes  **IWS response to specific drugs:** yes  **Sedatives weaned first:** yes |
| 19 | Purivatra et al. [22]  2021  Retrospective, observational, single center  Mixed ICU | Clonidine (enteral). Two groups: low-dose and high-dose  **IWS Incidence:**  Low dose vs. High dose:  17.5%-69.5% | Defined arbitrarily  New-­onset withdrawal symptoms defined as rebound hypertension (SBP >160 mmHg) and rebound tachycardia (HR >100 BPM)  **IWS as a secondary outcome** | **No. of signs and symptoms required:** 2  **Time of exposure:** median 5 [2-8]  **Defines specific signs and symptoms:** yes  **Differential diagnosis:** yes (at clinician’s discretion)  **α-2 agonist regimen specified:** yes  **Concomitant hypnotic/opioid use**: yes  **Defines α-2 agonist weaning regimen:** partially  **Evaluation time:** yes, up to 72 hours  **Exclusion criteria:** yes  **IWS response to specific drugs:** not specified (rescue drugs?)  **Sedatives/opioids weaned first:** mixed |
| 20 | Pathan et al. [23]  2021  Retrospective, observational, bicentric  Mixed ICU | Dexmedetomidine  **IWS Incidence:**  50/165 (30.3%) | Arbitrarily defined  Two or more of the following signs and symptoms in the 24 h of dexemedetomidine discontinuation: tachycardia, hypertension, nausea and/or vomiting, or agitation.  **IWS as a primary outcome** | **No. of signs and symptoms required:** ≥2  **Time of exposure:** ≥24 hours  **Defines specific signs and symptoms:** yes  **Differential diagnosis:** yes  **α-2 agonist regimen specified:** yes  **Concomitant hypnotic/opioid use**: yes  **Defines α-2 agonist weaning regimen:** yes  **Evaluation time:** yes, up to 24 hours  **Exclusion criteria:** yes  **IWS response to specific drugs:** yes  **Sedatives/opioids weaned first:** concomitantly |
| 21 | Hughes et al. [24]  2021  Double-blind, multicentric, RCT  Sepsis patients under MV | Propofol  **IWS Incidence:** 36/208 (17%)  Dexmedetomidine  **IWS Incidence:** 22/214 (10%) | Defined in supplementary material as tachycardia and diaphoresis present on the same daily assessment  Authors used the same criteria for both drugs.  **IWS a secondary outcome (safety endpoint)** | **No. of signs and symptoms required:** 2 out of 2 listed  **Time of exposure:** median 3 [2,6]  **Defines specific signs and symptoms:** yes  **Differential diagnosis:** not specified  **α-2 agonist/hypnotic regimen specified:** yes  **Concomitant opioid use**: yes  **Defines α-2 agonist/propofol weaning regimen:** yes  **Evaluation time:** yes, 48 hours  **Exclusion criteria:** yes  **IWS response to specific drugs:** not specified (rescue protocol)  **Sedatives/opioids weaned first:** not specified |
| 22 | Fetters et al. [25]  2022  Retrospective, single center  Mixed ICU | Dexmedetomidine  **IWS Incidence:** 2/105 (2%) | Based on daily provider progress note indicating dexmedetomidine withdrawal, as the objective symptoms of withdrawal (tachycardia, insomnia, hypertension, agitation, etc.) may also be present in other disease state processes in critically ill patients.  **IWS as a secondary outcome** | **No. of signs and symptoms required:** not specified  **Time of exposure:** not clear, but at least 72 hours  **Defines specific signs and symptoms:** no  **Differential diagnosis:** yes (at clinician’s discretion)  **α-2 agonist regimen specified:** yes  **Concomitant hypnotic/opioid use**: no  **Defines α-2 agonist weaning regimen:** no  **Evaluation time:** yes, up to 72 hours  **Exclusion criteria:** yes  **IWS response to specific drugs:** not specified  **Sedatives/opioids weaned first:** not applicable |
| 23 | Fox et al. [26]  2023  Prospective  Medical ICU patients | Opioids  (reported in fentanyl equivalents)  **IWS Incidence:**   \| 32/92 (35%) \| COWS \| \| --- \| --- \| \| 27/92 (29%) \| DSM-V \| | DSM-5 for IWS frequency  COWS for IWS severity  **Primary outcome:** moderate or severe IWS based on COWS.  **Secondary outcome:** prevalence of IWS of any severity based on COWS and prevalence of IWS based on DSM-5 | **No. of signs and symptoms required:** as per DSM-5 for IWS frequency and as per COWS for IWS severity  **Time of exposure:** ≥24 hours  **Defines specific signs and symptoms:** no  **Differential diagnosis:** no  **Opioid regimen specified:** no  **Concomitant hypnotic use**: yes  **Defines opioid weaning regimen:** no  **Evaluation time:** once daily, next calendar day after opioid cessation, up to 5 days or until severe IWS was developed  **Exclusion criteria:** yes  **IWS response to specific drugs:** no  **Sedatives weaned first:** yes |
| 24 | Maffei et al. [27]  2023  Retrospective  COVID-19 patients with ARDS under MV | Opioids  Hypnotics  All opioid and benzodiazepines converted in IV morphine and lorazepam equivalents  **IWS Incidence:** 57/115 (49.5%) | **Primary outcome:** IWS diagnosis in mechanically ventilated COVID-19 ARDS patients  Diagnosed in patients with COVID-19 ARDS, under MV, IV analgesia and sedation ≥5 days and classified as IWS and non-IWS based on receipt of scheduled oral sedative/analgesic regimens after cessation of IV therapy. | **No. of signs and symptoms required:** not specified  **Time of exposure:** ≥5 days  **Defines specific signs and symptoms:** no  **Differential diagnosis:** no  **Opioid regimen specified:** no  **Concomitant hypnotic use**: yes  **Defines opioid weaning:** no  **Evaluation time:** no  **Exclusion criteria:** yes  **IWS response to specific drugs:** yes  **Sedatives weaned first:** not specified |
| ANS – autonomic nervous system; ARDS – Acute Respiratory Distress Syndrome; AWS – acute withdrawal syndrome; CAM-ICU – Confusion Assessment Method for the Intensive Care Unit; CG – control group; CNS – central nervous system; COVID-19 – Coronavirus Disease-2019; COWS - Clinical Opiate Withdrawal Scale; DBP – diastolic blood pressure; DSM-V - Diagnostic and Statistical Manual of Mental Disorders 5^th^ Edition; ICU – intensive care unit; IWS - Iatrogenic Withdrawal Syndrome; MG – methadone group; MV – mechanical ventilation; RASS – Richmond Agitation and Sedation Scale; RCT – randomized controlled trial; SBP – systolic blood pressure; WAT-1 - Withdrawal Assessment Tool-1; | | | | |

**References**

[1] Cammarano WB, Pittet JF, Weitz S, Schlobohm RM, Marks JD. Acute withdrawal syndrome related to the administration of analgesic and sedative medications in adult intensive care unit patients. Crit Care Med 1998;26:676–84. https://doi.org/10.1097/00003246-199804000-00015.

[2] Himmelsbach Clifton Keck. Studies of certain addiction characteristics of (a) Dihydromorphine (“ Paramorphan”),(b) Dihydrodesoxymorphine-D (“ Desomorphine”),(c) Dihydrodesoxycodeine-D (“ Desocodeine”), and (d) Methyldihydromorphinone (“ Metopon”). Journal of Pharmacology and Experimental Therapeutics 1939;67:239–49.

[3] George CF, Robertson D. Clinical Consequences of Abrupt Drug Withdrawal. Med Toxicol 1987;2:367–82. https://doi.org/10.1007/BF03259954.

[4] Hofbauer R, Tesinsky P, Hammerschmidt V, Kofler J, Staudinger T, Kordova H, et al. No reduction in the sufentanil requirement of elderly patients undergoing ventilatory support in the medical intensive care unit. Eur J Anaesthesiol 1999;16:702–7. https://doi.org/10.1046/J.1365-2346.1999.00569.X.

[5] Korak-Leiter M, Likar R, Oher M, Trampitsch E, Ziervogel G, Levy J V., et al. Withdrawal following sufentanil/propofol and sufentanil/midazolam. Sedation in surgical ICU patients: correlation with central nervous parameters and endogenous opioids. Intensive Care Med 2005;31:380–7. https://doi.org/10.1007/S00134-005-2579-3.

[6] Jasinski DR. Chemotherapy of Addiction — A Pharmacological Perspective. Drug Dependence and Alcoholism, Boston, MA: Springer US; 1981, p. 1015–8. https://doi.org/10.1007/978-1-4684-3614-3_117.

[7] Liatsi D, Tsapas B, Pampori S, Tsagourias M, Pneumatikos I, Matamis D. Respiratory, metabolic and hemodynamic effects of clonidine in ventilated patients presenting with withdrawal syndrome. Intensive Care Med 2009;35:275–81. https://doi.org/10.1007/S00134-008-1251-0.

[8] Nseir S, Hoel J, Grailles G, Soury-Lavergne A, Di Pompeo C, Mathieu D, et al. Remifentanil discontinuation and subsequent intensive care unit-acquired infection: a cohort study. Crit Care 2009;13:R60. https://doi.org/10.1186/CC7788.

[9] Riker RR, Shehabi Y, Bokesch PM, Ceraso D, Wisemandle W, Koura F, et al. Dexmedetomidine vs Midazolam for Sedation of Critically Ill Patients: A Randomized Trial. JAMA 2009;301:489–99. https://doi.org/10.1001/JAMA.2009.56.

[10] Wanzuita R, Poli-de-Figueiredo LF, Pfuetzenreiter F, Cavalcanti AB, Westphal GA. Replacement of fentanyl infusion by enteral methadone decreases the weaning time from mechanical ventilation: a randomized controlled trial. Crit Care 2012;16. https://doi.org/10.1186/CC11250.

[11] Jakob SM, Ruokonen E, Grounds RM, Sarapohja T, Garratt C, Pocock SJ, et al. Dexmedetomidine vs midazolam or propofol for sedation during prolonged mechanical ventilation: two randomized controlled trials. JAMA 2012;307:1151–60. https://doi.org/10.1001/JAMA.2012.304.

[12] Ozaki M, Takeda J, Tanaka K, Shiokawa Y, Nishi S, Matsuda K, et al. Safety and efficacy of dexmedetomidine for long-term sedation in critically ill patients. J Anesth 2014;28:38–50. https://doi.org/10.1007/S00540-013-1678-5.

[13] Gagnon DJ, Riker RR, Glisic EK, Kelner A, Perrey HM, Fraser GL. Transition from dexmedetomidine to enteral clonidine for ICU sedation: an observational pilot study. Pharmacotherapy 2015;35:251–9. https://doi.org/10.1002/PHAR.1559.

[14] Sandiumenge A, Torrado H, Muñoz T, Alonso M, Jiménez MJ, Alonso J, et al. Impact of harmful use of alcohol on the sedation of critical patients on mechanical ventilation: A multicentre prospective, observational study in 8 Spanish intensive care units. Med Intensiva 2016;40:230–7. https://doi.org/10.1016/J.MEDIN.2015.06.008.

[15] Wang PP, Huang E, Feng X, Bray CA, Perreault MM, Rico P, et al. Opioid-associated iatrogenic withdrawal in critically ill adult patients: a multicenter prospective observational study. Ann Intensive Care 2017;7. https://doi.org/10.1186/S13613-017-0310-5.

[16] Bouajram RH, Bhatt K, Croci R, Baumgartner L, Puntillo K, Ramsay J, et al. Incidence of Dexmedetomidine Withdrawal in Adult Critically Ill Patients: A Pilot Study. Crit Care Explor 2019;1:E0035. https://doi.org/10.1097/CCE.0000000000000035.

[17] Capilnean A, Martone A, Rosu VA, Sandu PR, Murgoi P, Frenette AJ, et al. Validation of the Withdrawal Assessment Tool-1 in Adult Intensive Care Patients. Am J Crit Care 2019;28:361–9. https://doi.org/10.4037/AJCC2019559.

[18] Bhatt K, Thompson Quan A, Baumgartner L, Jia S, Croci R, Puntillo K, et al. Effects of a Clonidine Taper on Dexmedetomidine Use and Withdrawal in Adult Critically Ill Patients-A Pilot Study. Crit Care Explor 2020;2:E0245. https://doi.org/10.1097/CCE.0000000000000245.

[19] Arroyo-Novoa CM, Figueroa-Ramos MI, Balas M, Rodríguez P, Puntillo KA. Opioid and Benzodiazepine Withdrawal Syndromes in Trauma ICU Patients: A Prospective Exploratory Study. Crit Care Explor 2020;2:E0089. https://doi.org/10.1097/CCE.0000000000000089.

[20] Hyun D gon, Huh JW, Hong SB, Koh Y, Lim CM. Iatrogenic Opioid Withdrawal Syndrome in Critically Ill Patients: a Retrospective Cohort Study. J Korean Med Sci 2020;35. https://doi.org/10.3346/JKMS.2020.35.E106.

[21] Taesotikul S, Dilokpattanamongkol P, Tangsujaritvijit V, Suthisisang C. Incidence and clinical manifestation of iatrogenic opioid withdrawal syndrome in mechanically ventilated patients. Curr Med Res Opin 2021;37:1213–9. https://doi.org/10.1080/03007995.2021.1928616.

[22] Purivatra E, Guenette M, Coleman B, Cheung A, Burry L. High-versus low-dose clonidine for sedation and analgesia in critically ill adults: A retrospective cohort study. J Clin Pharm Ther 2021;46:1706–13. https://doi.org/10.1111/JCPT.13523.

[23] Pathan S, Kaplan JB, Adamczyk K, Chiu SH, Shah C V. Evaluation of dexmedetomidine withdrawal in critically ill adults. J Crit Care 2021;62:19–24. https://doi.org/10.1016/J.JCRC.2020.10.024.

[24] Hughes CG, Mailloux PT, Devlin JW, Swan JT, Sanders RD, Anzueto A, et al. Dexmedetomidine or Propofol for Sedation in Mechanically Ventilated Adults with Sepsis. N Engl J Med 2021;384:1424–36. https://doi.org/10.1056/NEJMOA2024922.

[25] Fetters MB, Diep C, Ran R, Kloosterboer A. Effect of Enteral Guanfacine on Dexmedetomidine Use in the ICU. Crit Care Explor 2022;4:E0785. https://doi.org/10.1097/CCE.0000000000000785.

[26] Fox MA, Carothers C, Dircksen KK, Birrer KL, Choi MJ, Mukkera SR. Prevalence and Risk Factors for Iatrogenic Opioid Withdrawal in Medical Critical Care Patients. Crit Care Explor 2023;5:E0904. https://doi.org/10.1097/CCE.0000000000000904.

[27] Maffei MV, Laehn S, Bianchini M, Kim A. Risk Factors Associated With Opioid/Benzodiazepine Iatrogenic Withdrawal Syndrome in COVID-19 Acute Respiratory Distress Syndrome. J Pharm Pract 2023;36:1362–9. https://doi.org/10.1177/08971900221116178.
